# Alginate-Based Solid Foam Incorporating Rügen Chalk: A Novel Platform for Modern Application of Peloids

**DOI:** 10.3390/ph19070973

**Published:** 2026-06-23

**Authors:** Mantas Jurkonis, Modestas Žilius, Karolis Banionis, Elena Jasiūnienė, Jurga Bernatoniene

**Affiliations:** 1 Institute of Pharmaceutical Technologies, Lithuanian University of Health Sciences, LT-50162 Kaunas, Lithuania; mantas.jurkonis@lsmu.lt (M.J.); modestas.zilius@lsmu.lt (M.Ž.); 2 Department of Clinical Pharmacy, Lithuanian University of Health Sciences, LT-50161 Kaunas, Lithuania; 3 Institute of Architecture and Construction, Kaunas University of Technology, LT-44405 Kaunas, Lithuania; karolis.banionis@ktu.lt; 4 Ultrasound Research Institute, Kaunas University of Technology, LT-51368 Kaunas, Lithuania; elena.jasiuniene@ktu.lt; 5 Department of Electronics Engineering, Kaunas University of Technology, LT-51368 Kaunas, Lithuania; 6 Department of Drug Technology and Social Pharmacy, Lithuanian University of Health Sciences, LT-50161 Kaunas, Lithuania

**Keywords:** Rügen chalk, calcium carbonate, alginate, solid foam, microcomputed tomography, pH, thermal retention, balneotherapy, topical formulation

## Abstract

**Background/Objectives:** Natural calcium carbonate materials such as Rügen chalk have a long history of use in balneology and rehabilitation, particularly for musculoskeletal disorders, yet their application remains largely confined to traditional, labour-intensive forms such as powders, suspensions, and packs, which limit usability and broader clinical translation. This study aimed to develop an alginate-based solid foam incorporating Rügen chalk and to evaluate how key formulation components influence its structural, mechanical, and thermal properties relevant for therapeutic use. **Methods:** Alginate–chalk foams were prepared by mechanical mixing of a sodium alginate–Rügen chalk paste with an amino acid-based surfactant, while in situ CO_2_ generation from D–glucono–δ–lactone (GDL) induced calcium-mediated alginate gelation and foam stabilization. A central composite design with response surface methodology was used to assess the effects of alginate, chalk, and Perlastan^®^–GDL content on foam pH, overrun, firmness, springiness, pore volume, sphericity, pore density, specific internal surface area, and heat-loss time. Foam microstructure was characterized by optical microscopy and microcomputed tomography (µCT), and the thermal conductivity and cooling behaviour of the selected formulation were compared with therapeutic peat. **Results:** Stable, elastic solid foams with a three-dimensional porous architecture were obtained across the investigated composition range. Foam overrun (30.8–57.1%) was primarily governed by sodium alginate and Rügen chalk concentrations, while firmness (7.4–15.2 N) increased predominantly with alginate content, and springiness remained high (70–78%), indicating good elastic recovery. Response surface modelling and ANOVA confirmed sodium alginate as the dominant factor influencing both mechanical and structural properties, with statistically significant effects on overrun, firmness, springiness, heat loss, porosity, and specific internal surface. µCT analysis revealed that all foam formulations were predominantly composed of fine, closed-cell pores, with over 96% of pores having volumes below 0.5 mm^3^ and a consistent median pore volume of 0.02 mm^3^. Structural differences between formulations were governed primarily by pore number and spatial distribution rather than pore size. Strong correlations were identified between µCT-derived parameters, particularly between specific internal surface, porosity, and pore density, confirming that internal architecture is controlled by pore population rather than individual pore dimensions. Thermal analysis demonstrated that the optimized formulation exhibited thermal conductivity comparable to therapeutic peat and maintained clinically relevant temperatures (35–45 °C) for more than one hour. Based on predefined performance criteria (overrun ≥ 50%, firmness ≤ 10 N, heat loss ≥ 120 s), formulation 7 was identified as optimal, combining favourable mechanical properties, structural uniformity and thermal retention. **Conclusions:** Alginate-based solid foams incorporating Rügen chalk constitute a feasible and tunable platform that combines efficient mineral loading, elastic porosity, and effective heat retention, offering a practical and modern alternative to conventional mineral-based therapeutic applications in balneology and rehabilitation.

## 1. Introduction

Natural therapeutic factors derived from geological sources have been used for centuries in balneology, rehabilitation medicine, and supportive healthcare, where mineral–based materials are valued for their physicochemical stability, high purity, and generally favourable safety profiles [1,2]. In particular, peloids and carbonate-based minerals have played an important role in the treatment of musculoskeletal disorders, chronic pain syndromes, and inflammatory skin diseases, especially within spa and rehabilitation medicine [3,4]. Recent clinical evidence has confirmed that balneotherapy and peloid therapy effectively reduce pain and improve the functional state of patients with knee joint osteoarthritis [5], demonstrating significant improvements in walking speed, joint mobility, and physical activity compared to conventional physical therapy alone [6]. Despite their long-standing empirical use and documented patient acceptance, many natural mineral materials are still applied in traditional forms—such as powders, slurries, packs, or baths—that limit usability and hinder their integration into modern pharmaceutical formulation strategies [7].

Low back pain and other musculoskeletal disorders represent a major public health burden with considerable socioeconomic impact, accounting for a substantial proportion of disability, reduced quality of life, and healthcare costs in industrialized countries [8]. Within this context, non-pharmacological and supportive therapies, including mineral-based treatments, remain an important component of multidisciplinary rehabilitation concepts [9]. Clinical observations and patient-reported outcomes from spa and rehabilitation settings indicate that natural mineral applications, including carbonate-based peloids, are particularly valued for the management of muscle tension, joint pain, degenerative joint diseases, and chronic inflammatory conditions of the locomotor system [6,10].

Rügen chalk (German: *Rügener Heilkreide*) is a naturally occurring calcium carbonate (CaCO_3_) material of Late Cretaceous (Maastrichtian) origin, formed as a pelagic carbonate sediment from biogenic marine sources and widely exposed on the island of Rügen, Germany [11,12,13]. The chalk deposits reach thicknesses exceeding 90 m and represent one of the most comprehensively studied Upper Cretaceous chalk localities in Europe [12,13]. The material consists predominantly of calcium carbonate (approximately 98%), with minor contributions of magnesium carbonate, siliceous components, iron and aluminum compounds, as well as trace mineral constituents such as phosphorus and sulfur [14]. As a fine-grained, microcrystalline material, Rügen chalk is characterized by particle sizes of approximately 1–2 µm and a specific surface area of 5–6 m^2^/g, as determined by BET analysis [15]. It exhibits alkaline surface chemistry, a density of approximately 2.74 g/cm^3^, low water solubility (~16 mg/L at 20 °C), and a pH range of 7–10 in saturated aqueous suspension [15]. Safety evaluations and application observations have demonstrated that Rügen chalk contains no hazardous constituents, shows no acute or chronic toxicity, and exhibits excellent skin tolerability, with no evidence of allergenic potential in epicutaneous testing [15].

Traditionally, Rügen chalk has been applied in powdered form or as aqueous suspensions, packings, or baths within spa and rehabilitation settings. These applications have been associated with reported benefits in muscle tension, joint pain, rheumatic conditions, chronic arthritis, lymphatic congestion, and selected inflammatory skin diseases [16]. However, natural peloids and mineral suspensions are inherently complex multiphase systems with variable mineralogical, chemical, and microbiological composition, which complicates standardization of active principles, quality control, and sanitary safety [17]. Consequently, traditional application forms are often labor-intensive, require professional supervision, and necessitate removal after use, which limits their feasibility outside specialized facilities and reduces continuity of therapy in the home environment [3]. These practical constraints substantially restrict the broader translation of natural mineral therapeutic factors into contemporary, patient-oriented product formats and pharmaceutical contexts.

Recent advances in pharmaceutical technology and biomaterials research have highlighted the potential of mineral–polymer composite systems, particularly those combining calcium carbonate with naturally derived polymers [18]. Alginate, a biocompatible and biodegradable polysaccharide widely used in pharmaceutical and biomedical applications, forms elastic three-dimensional networks under mild conditions and is capable of immobilizing particulate mineral phases within a cohesive matrix [19,20]. Previous studies have demonstrated that alginate–CaCO_3_ systems can be engineered into structured materials with controlled porosity, mechanical integrity, and stable mineral incorporation [21,22,23]. Recent investigations have shown that alginate-mediated crystallization enables the formation of nanoscale calcium carbonate phases within hydrogel networks, offering tunable mineral dimensions, shapes, and polymorphic forms—a highly adaptable approach for creating structural composite materials with biomimetic properties [24]. Moreover, freeze-cast alginate–calcium carbonate composite scaffolds have demonstrated the ability to control pore size, wall thickness, and mechanical properties through in situ cross-linking by calcium ions released from the mineral phase, presenting attractive opportunities for tissue engineering and biomedical applications [25].

Solid foam architectures represent an emerging class of materials that combine porosity, elasticity, and structural stability within a self-supporting three-dimensional network [26,27]. Recent work has shown that alginate-based hydrogel foams can be generated in a single step via CO_2_-induced simultaneous foaming and gelation, enabling precise control of gas fraction and foam stability [28]. Unlike liquid or sprayable foams, elastic solid foams with a gelatinous consistency can retain mineral components without requiring post-application removal while preserving physicochemical characteristics comparable to those of the native mineral material [29]. High-pressure foaming and solid-state foaming techniques have been widely investigated for fabricating biocompatible and biodegradable porous scaffolds, offering fine control over pore architecture and material properties at low processing temperatures [26,30]. Such systems are particularly attractive for therapeutic applications, as they allow prolonged skin contact, controlled mechanical properties, and improved user acceptance while minimizing handling complexity.

Despite these advances, few studies have attempted to integrate traditional therapeutic minerals, such as Rügen chalk, into modern foam-based formulation platforms that combine the functional benefits of the natural mineral with the practical advantages of a stable, porous, and elastic matrix. Furthermore, while alginate–calcium carbonate composites have been extensively studied for biomedical scaffolds and tissue engineering, their application in balneotherapy and topical therapeutic formulations remains largely unexplored. The lack of systematic investigations into the physicochemical and thermal properties of such systems represents a significant gap in the development of next-generation mineral-based therapeutic products.

The aim of this study was therefore to integrate a natural therapeutic mineral factor—Rügen chalk—into an alginate-based solid foam system; to systematically characterize its physicochemical, mechanical, and thermal properties using response surface methodology; and to evaluate whether this novel formulation preserves the key functional characteristics of the native mineral while offering improved convenience, usability, and applicability beyond traditional spa-based settings. This work demonstrates that elastic solid foams composed of alginate and natural calcium carbonate represent a viable and innovative platform for the modern application of natural mineral-based therapeutic materials, with potential implications for home-based therapy and broader clinical accessibility.

## 2. Results

### 2.1. Effect of Formulation Composition on Foam Overrun

**Figure 1:**
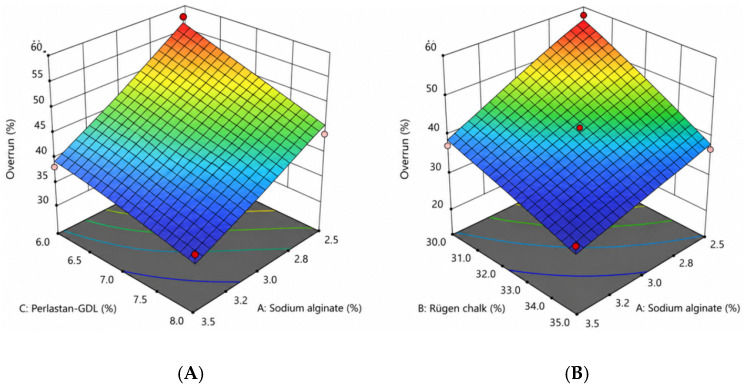
Effect of formulation variables on foam overrun (%): (**A**) combined influence of sodium alginate and Perlastan^®^–GDL level at fixed Rügen chalk concentration of 30.0%; (**B**) combined influence of sodium alginate concentration and Rügen chalk content at fixed Perlastan^®^–GDL concentration of 6.0%. Red dots represent experimental data points, while contour lines and colour gradients indicate the predicted response surface.

The foam overrun values in this study ranged from 30.8% to 57.1%, indicating a clear dependence on formulation composition. The lowest overrun was observed in the foam containing 3.5% sodium alginate, 35% Rügen chalk, and a 6% Perlastan^®^–GDL mixture, whereas the highest overrun was recorded for the foam containing 2.5% sodium alginate, 30% Rügen chalk, and 6% Perlastan^®^–GDL mixture. The influence of sodium alginate concentration, Rügen chalk content, and Perlastan^®^–GDL level on foam expansion is illustrated in Figure 1.

According to the regression model in coded variables Overrun=38.29−5.40A−3.97B−1.60C+2.43AB+2.78BC (where A—sodium alginate; B—Rügen chalk; C—Perlastan^®^–GDL), the magnitude of the individual effects on overrun increases in the following order: C < B < A. This finding is consistent with the response surface plots in Figure 1, which show a stronger influence of alginate concentration compared with chalk and Perlastan^®^–GDL levels on foam expansion.

### 2.2. Optical Microscopy of Alginate–Rügen Chalk Foams

**Figure 2:**
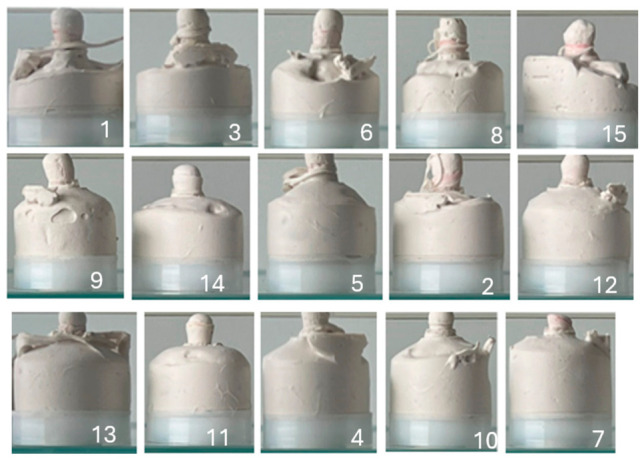
Macroscopic appearance of alginate–Rügen chalk foams arranged in ascending order of overrun (from left to right and top to bottom).

Macroscopic inspection (Figure 2) revealed marked differences in foam expansion, with low-overrun samples forming compact, flat surface structures and high-overrun formulations appearing more voluminous, with softer contours and dome surfaces.

**Figure 3:**
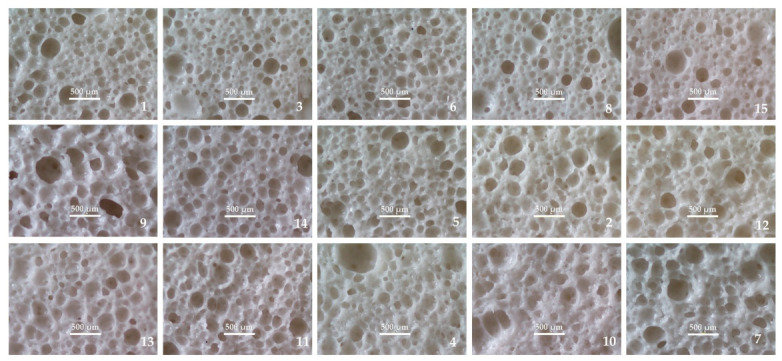
Optical microscopy images of alginate–Rügen chalk foams arranged in ascending order of overrun (from left to right and top to bottom). The scale bar shown in each micrograph corresponds to 500 µm.

To elucidate the structural basis of these differences, cross-sectional micrographs were acquired under low magnification with side illumination (Figure 3). All foams exhibited a three-dimensional porous architecture characteristic of gas-expanded polymeric systems formed by in situ CO_2_ generation during gelation. Representative optical cross-sections illustrating predominantly fine, closed-cell pores with formulation-dependent variations in the proportion of larger voids.

Low-overrun foams (overrun < 30%) displayed a dense microstructure with predominantly small, closed pores and relatively thick walls, indicating limited porosity and a compact, mechanically constrained architecture. Formulations with intermediate overrun (approximately 30–50%) showed a more homogeneous, sponge-like morphology with uniformly distributed, rounded pores and a narrow size range, forming a continuous and elastic three-dimensional network. High-overrun foams (>50%) exhibited pronounced pore coalescence, larger and more irregular voids, and locally thinned walls, resulting in a less uniform microstructure indicative of excessive gas expansion during gelation.

Overall, increasing overrun was associated with a gradual increase in pore size and bulk porosity, confirming that the extent of CO_2_ generation and retention during gelation is a key determinant of foam morphology. Formulations with intermediate overrun values exhibited a microarchitecture that combined relatively high porosity with preserved structural integrity and an elastic, sponge-like texture, rather than a brittle or highly collapsible structure.

### 2.3. Microcomputed Tomography (µCT) Analysis

**Figure 4:**
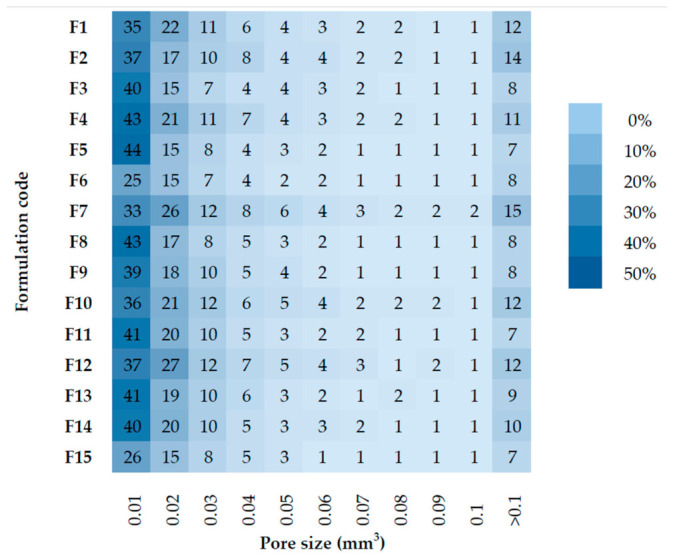
Heatmap showing the percentage distribution of pore volume classes in alginate–Rügen chalk foam formulations. The majority of pores were concentrated within the 0.01–0.03 mm^3^ range, while pore volume fractions above 0.10 mm^3^ represented only a minor proportion of the total pore population.

Quantitative X-ray microcomputed tomography (µCT) data revealed a consistent structural pattern across all samples, characterized by a predominance of low-volume voxels and only a small fraction of high-volume domains. The pore volume median was 0.02 mm^3^ in all formulations. Across all formulations, pore volumes between 0.01 and 0.03 mm^3^ represented the dominant fraction of the pore population, accounting for approximately 48–59% of all detected pores. The proportion of pores larger than 0.10 mm^3^ ranged from 7% to 15% depending on the formulation. These results indicate that the foam structure is predominantly composed of fine pores, with only a limited contribution from larger pore domains. A heatmap illustrating the percentage distribution of pore volume classes across all formulations is presented in Figure 4.

**Figure 5:**
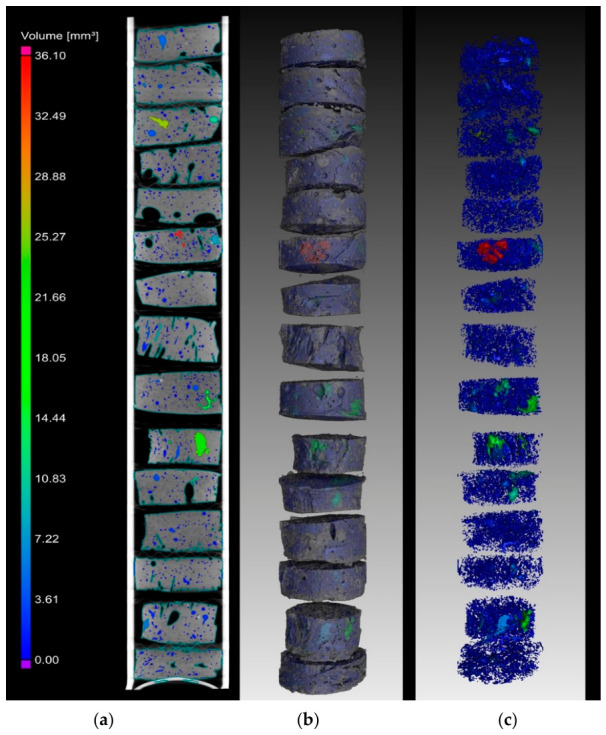
X and y microcomputed tomography (µCT) analysis of the developed foam samples. The samples are arranged vertically from bottom to top according to the experimental run order (formulation codes 1 to 15): (**a**) 2D reconstructions of the foam samples; (**b**) 3D reconstructions of the foam samples; (**c**) voxel-based volume maps, where colour coding represents voxel volume, with blue indicating low-volume regions (fine pores or dense matrix) and red indicating high-volume domains (larger pores or voids).

None of the investigated formulations exhibited a fully developed open-cell structure. Although localized pore coalescence was observed in several samples, the resulting structures remained predominantly closed-cell, with only partial interconnectivity between pores. This indicates that, despite gas evolution and occasional bubble merging, the matrix viscosity and gelation kinetics were sufficient to prevent the formation of a continuous open pore network. Representative reconstructed 2D and 3D images and corresponding voxel-based volumetric distributions are presented in Figure 5.

The µCT-derived parameters revealed clear, systematic differences in the internal foam architecture, driven by changes in surface area, porosity, pore number and pore shape. The key quantitative µCT-derived structural parameters are summarized in Table 1.

The lowest specific internal surface (0.37 mm^−1^) was observed for the foam containing 3.0% sodium alginate, 32.5% Rügen chalk and 6% Perlastan^®^–GDL mixture (formulation 11), whereas the highest specific internal surface (1.03 mm^−1^) was recorded for the foam containing 2.5% sodium alginate, 32.5% Rügen chalk and 7% Perlastan^®^–GDL mixture (formulation 10).

Similarly, the lowest porosity (2.82%) was obtained for formulation 11 (3.0% sodium alginate, 32.5% Rügen chalk, and 6% Perlastan^®^–GDL), while the highest porosity (7.58%) was observed for formulation 10 (2.5% sodium alginate, 32.5% Rügen chalk, and 7% Perlastan^®^–GDL).

Mean pore volume values were relatively similar across all formulations, ranging from 0.04 to 0.06 mm^3^, indicating that differences between samples were driven primarily by pore number and morphology rather than by large shifts in the typical pore size.

The mean pore sphericity varied only moderately between formulations (0.79–0.90), suggesting that pores were predominantly near-spherical in all systems; the highest sphericity (0.90) was found in formulations 3, 8, and 15, whereas the lowest values (~0.79–0.80) were observed in formulations 2 and 11.

Pore density spanned from 0.63 to 1.40 pores/mm^3^, with the sparsest pore population in formulation 8 (3.5% sodium alginate, 35.0% Rügen chalk, and 8% Perlastan^®^–GDL) and the highest pore density in formulation 2 (2.5% sodium alginate, 35.0% Rügen chalk, and 8% Perlastan^®^–GDL), indicating that adjustments in alginate and chalk content primarily modulated the number of pores per unit volume rather than their individual shape.

Gas generation, viscosity changes and surfactant-mediated stabilization together likely cause the observed heterogeneity in bubble growth and pore structure.

### 2.4. Effect of Formulation Variables on Foam Firmness

**Figure 6:**
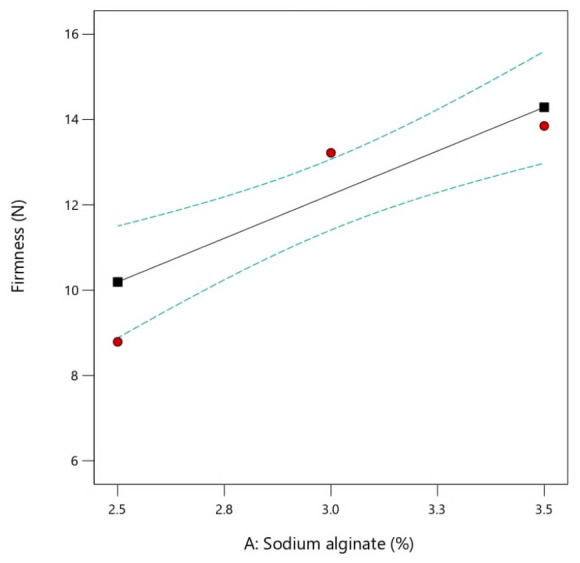
Firmness prediction plot for sodium alginate concentration. Red circles represent experimental values, black squares represent model-predicted values, the solid line indicates the fitted regression model, and dashed lines show the 95% confidence intervals.

Foam firmness values varied from approximately 7.4 to 15.2 N, indicating a pronounced dependence on formulation composition. The lowest firmness was observed in the foam containing 2.5% sodium alginate, 30% Rügen chalk, and 6% Perlastan^®^–GDL mixture, whereas the highest firmness was recorded for the foam containing 3.5% sodium alginate, 30% Rügen chalk, and 6% Perlastan^®^–GDL mixture. The effect of sodium alginate concentration on foam firmness, as predicted by the one-factor response surface, is illustrated in Figure 6.

The response surface analysis indicated that the sodium alginate concentration was the dominant factor affecting foam firmness. Increasing alginate content led to a clear and consistent increase in firmness, reflecting the formation of a denser and more mechanically resistant polymer network, likely due to enhanced chain entanglement and stronger gel structuring at higher concentrations. In contrast, Rügen chalk exerted a weaker but still positive effect, consistent with a moderate reinforcing action of the mineral phase within the alginate matrix.

According to the regression model in coded variables, Firmness=12.24+2.05A+0.211B−0.019C (where A—sodium alginate; B—Rügen chalk; C—Perlastan^®^–GDL), the magnitude of the individual effects on firmness increases in the following order: C < B < A.

From a practical perspective, formulations with higher alginate content and moderate-to-high chalk loading produced foams with a firmer, more shape-stable texture, whereas low-alginate systems yielded softer, less mechanically resistant structures.

### 2.5. Effect of Formulation Variables on Foam Springiness

**Figure 7:**
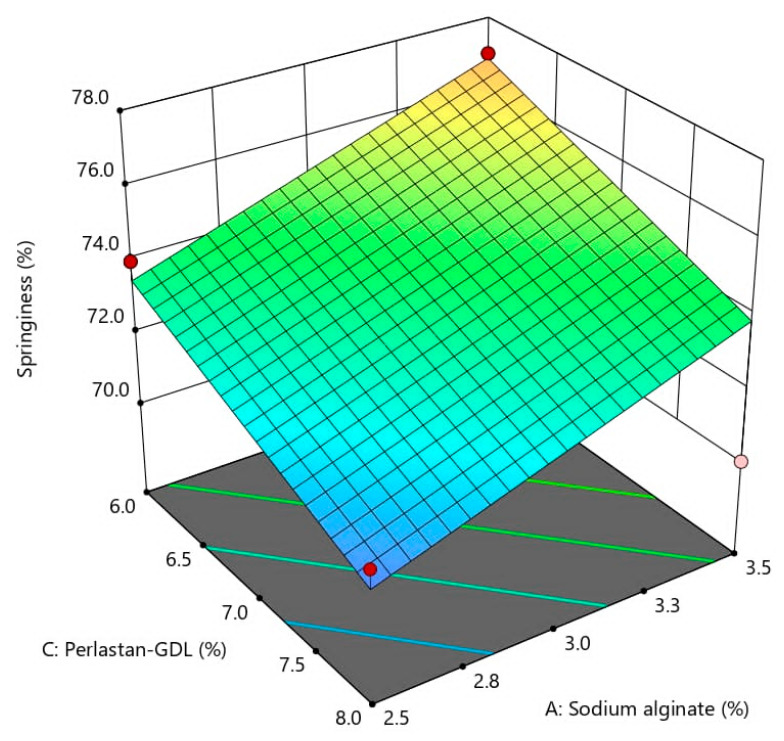
Response surface plot showing the effect of sodium alginate concentration (A) and Perlastan^®^–GDL ratio (C) on foam springiness at a fixed Rügen chalk concentration of 30.0%.

The springiness of the foams ranged from 70% to 78%, indicating consistently good elastic recovery across the tested formulations. The lowest springiness was observed in the foam containing 3.5% sodium alginate, 30% Rügen chalk, and 8% Perlastan^®^–GDL mixture, whereas the highest overrun was recorded for the foam containing 3.5% sodium alginate, 32.5% Rügen chalk, and 7% Perlastan^®^–GDL mixture. The influence of sodium alginate concentration, Rügen chalk content, and Perlastan^®^–GDL level on foam springiness is illustrated in Figure 7.

Response surface analysis showed that foam springiness was primarily influenced by sodium alginate and the Perlastan^®^–GDL system. Increasing sodium alginate content led to a clear enhancement of springiness, reflecting the formation of a more continuous and elastic polymeric network capable of recovering after deformation.

According to the regression model in coded variables Springiness=73.67+1.7A+0.8B−1.5C (where A—sodium alginate; B—Rügen chalk; C—Perlastan^®^–GDL), the magnitude of the individual effects on springiness increases in the order of B < C < A, confirming the dominant role of alginate compared with chalk and the surfactant–GDL system.

### 2.6. Effect of Formulation Variables on Foam pH

**Figure 8:**
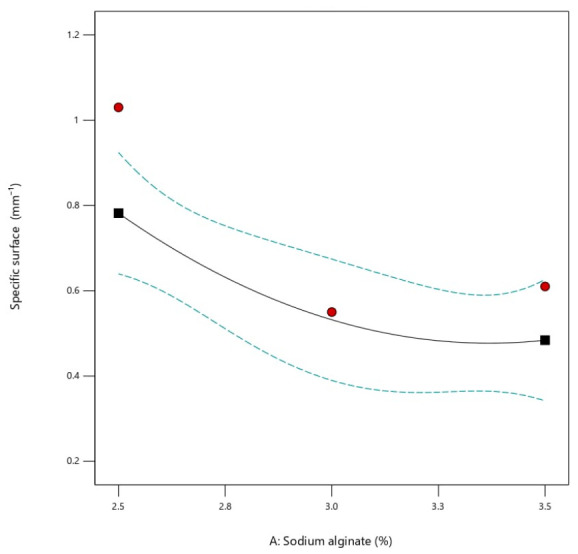
The specific internal surface prediction plot for sodium alginate concentration. Red circles represent experimental values, black squares represent model-predicted values, the solid line indicates the fitted regression model, and dashed lines show the 95% confidence intervals.

**Figure 9:**
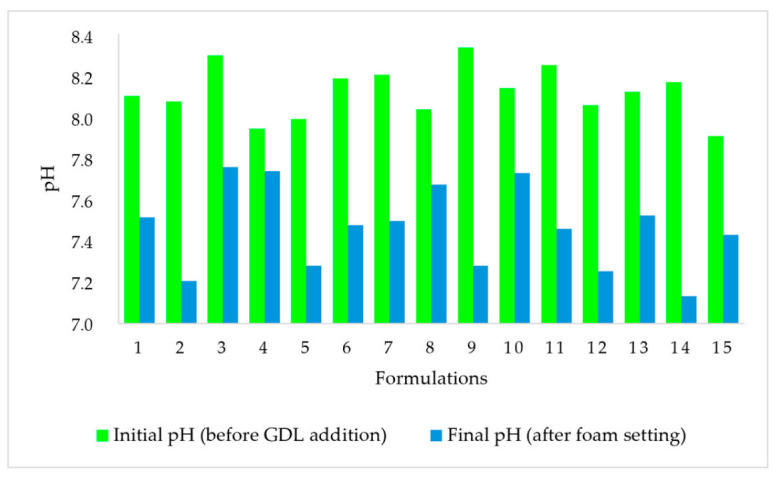
Comparison of initial pH (before GDL addition) and final pH (after foam setting) of alginate–Rügen chalk foam formulations.

The pH values of the alginate–Rügen chalk foams remained within a narrow near-neutral interval, ranging from 7.14 to 7.81 (mean ≈ 7.5), indicating that the investigated variations in sodium alginate, Rügen chalk and Perlastan^®^–GDL content did not markedly perturb the overall acid–base balance of the system (Figure 8). The initial pH of the alginate–chalk suspensions before GDL addition was moderately alkaline and highly consistent across all runs (7.97–8.43, mean ≈ 8.2), whereas GDL-mediated slow acidification reproducibly lowered the pH of the set foams by about 0.6–1.1 pH units into the near-neutral range (Figure 9).

The lowest final pH (7.14) was observed in the foam containing 3.5% sodium alginate, 30% Rügen chalk, and 6% Perlastan^®^–GDL mixture (formulation 14), whereas the highest final pH (7.81) was recorded for the foam containing 3.5% sodium alginate, 35% Rügen chalk, and 6% Perlastan^®^–GDL mixture (formulation 3). Across all formulations, the foams therefore maintained a near-neutral pH that is considered compatible with topical skin application, and the modest residual differences between compositions are unlikely to be clinically relevant.

### 2.7. Effect of Formulation Variables on Foam Heat Loss

**Figure 10:**
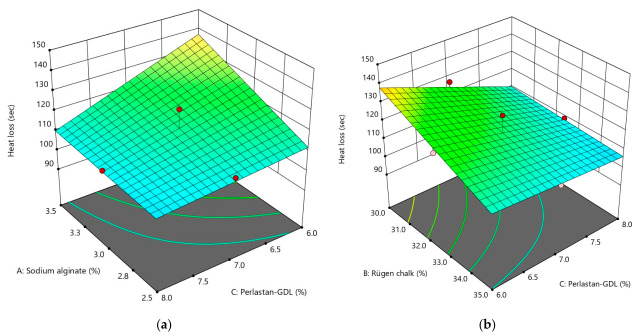
Three-dimensional response surface plots showing the combined effects of formulation variables on heat loss time of Rügen chalk-based alginate foams: (**a**) interaction between sodium alginate (A) and Perlastan^®^–GDL at fixed Rügen chalk concentration of 30%; (**b**) interaction between Rügen chalk (B) and Perlastan^®^–GDL (C) system at fixed sodium alginate concentration of 3.5%.

The heat loss time of the foams varied between 95 and 148 s across the investigated formulation domain, indicating notable differences in thermal retention behaviour. The shortest heat loss time was observed in the foam containing 2.5% sodium alginate, 35% Rügen chalk, and 6% Perlastan^®^–GDL mixture, whereas the longest heat loss was recorded for the foam containing 3.5% sodium alginate, 30% Rügen chalk, and 6% Perlastan^®^–GDL mixture. The influence of sodium alginate concentration, Rügen chalk content, and Perlastan^®^–GDL level on foam heat loss is illustrated in Figure 10.

Response surface analysis showed that Perlastan^®^–GDL content exerted the strongest positive effect on heat retention, with increasing Perlastan^®^–GDL concentration leading to a marked prolongation of heat loss time.

According to the regression model in coded variables Heat loss=116.53+7.2A−7.6B−8.2C+0.625AB−5.37AC+5.63BC (where A—sodium alginate; B—Rügen chalk; C—Perlastan^®^–GDL), the magnitude of the individual effects on heat loss increases in the following order: A < B < C. The combined effects of the formulation variables revealed notable interaction behavior. At lower Perlastan^®^–GDL levels, increasing sodium alginate substantially prolonged the heat loss time, whereas at higher Perlastan^®^–GDL concentrations, this effect was partially attenuated, as reflected by the curvature of the response surface. Similarly, the interaction between Rügen chalk and Perlastan^®^–GDL influenced heat loss, suggesting that mineral–polymer interactions and crosslinking intensity jointly govern thermal dissipation in the foams.

### 2.8. Effect of Formulation Variables on Foam Specific Internal Surface

The specific internal surface of the foams ranged from 0.37 to 1.03 mm^−1^ across the investigated formulation space, indicating marked differences in the internal pore–matrix interfacial area. The lowest specific internal surface (0.37 mm^−1^) was observed for the foam containing 3.0% sodium alginate, 32.5% Rügen chalk, and 6% Perlastan^®^–GDL mixture (formulation 11), whereas the highest specific internal surface (1.03 mm^−1^) was recorded for the foam containing 2.5% sodium alginate, 32.5% Rügen chalk, and 7% Perlastan^®^–GDL mixture (formulation 10). The influence of sodium alginate concentration, Rügen chalk content, and Perlastan^®^–GDL level on the foam’s specific internal surface is illustrated in Figure 10.

Response surface analysis showed that sodium alginate content exerted the strongest negative effect on the foam’s specific internal surface, with increasing alginate concentration leading to a marked decrease in the specific internal surface. According to the regression model in coded variables, Specific internal surface=0.532−0.149A+0.101A2 (where A—sodium alginate), this trend reflects the progressive thickening and stabilization of the polymer matrix at higher alginate levels.

### 2.9. Effect of Formulation Variables on Foam Porosity

**Figure 11:**
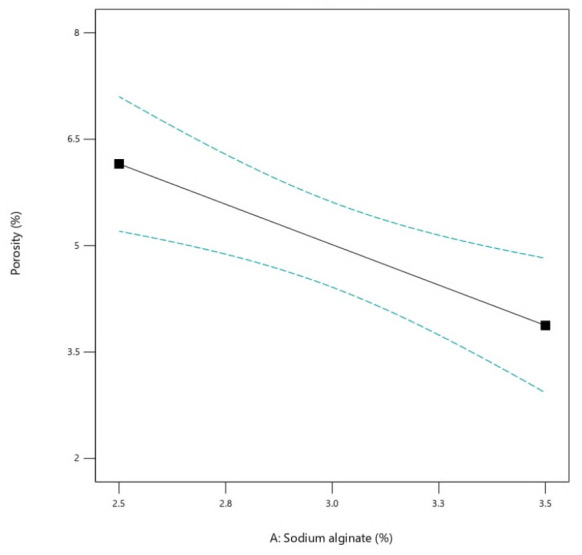
The specific internal surface prediction plot for foam porosity. Black squares represent model-predicted values, the solid line indicates the fitted regression model, and dashed lines show the 95% confidence intervals.

The porosity of the foams ranged from 2.82% to 7.58% within the investigated formulation space, confirming that all systems exhibited a relatively low but clearly distinguishable gas phase fraction. The lowest porosity (2.82%) was obtained for the foam containing 3.0% sodium alginate, 32.5% Rügen chalk and 6% Perlastan^®^–GDL mixture (formulation 11), whereas the highest porosity (7.58%) was observed for the foam containing 2.5% sodium alginate, 32.5% Rügen chalk and 7% Perlastan^®^–GDL mixture (formulation 10). The influence of sodium alginate concentration on foam porosity is illustrated in Figure 11.

One-factor response surface analysis showed that sodium alginate content exerted a clear negative effect on porosity, with increasing alginate concentration leading to a gradual decrease in the pore volume fraction. According to the regression model in coded variables, Porosity=5.01−1.14A (where A—sodium alginate), this behaviour reflects the higher viscosity and faster gelation at elevated alginate levels, which limit bubble expansion and thus reduce the overall gas volume entrapped in the foams.

### 2.10. Effect of Formulation Variables on Foam Pore Density

**Figure 12:**
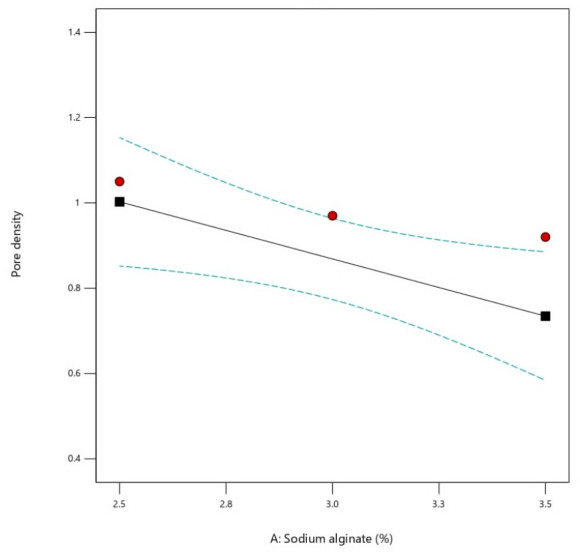
Pore density prediction plot for sodium alginate concentration. Red circles represent experimental values, black squares represent model-predicted values, the solid line indicates the fitted regression model, and dashed lines show the 95% confidence intervals.

Foam pore density analysis showed that formulation variables also had a pronounced effect on the number of pores per unit volume. Across the investigated formulation space, pore density ranged from 0.63 to 1.40 pores/mm^3^, indicating substantial differences in the fineness of the cellular structure. The sparsest pore population (0.63 pores/mm^3^) was observed for the foam containing 3.5% sodium alginate, 35.0% Rügen chalk, and 8% Perlastan^®^–GDL mixture (formulation 8), whereas the highest pore density (1.40 pores/mm^3^) was obtained for the foam containing 2.5% sodium alginate, 35.0% Rügen chalk, and 8% Perlastan^®^–GDL mixture (formulation 2). The influence of sodium alginate concentration on pore density is illustrated in Figure 12.

One-factor response surface analysis revealed that increasing alginate content led to a progressive reduction in pore density, consistent with a more viscous and rapidly gelling matrix that restricts bubble nucleation and growth. According to the regression model in coded variables Pore density=0.8687−0.134A (where A—sodium alginate), alginate concentration primarily controls pore formation density.

### 2.11. Statistical Analysis of Response Models via ANOVA

The adequacy and statistical significance of the developed response surface models were evaluated using analysis of variance (ANOVA). Model fit was assessed based on the overall model *p*-value (significant at *p* < 0.05), together with adjusted and predicted R^2^ values and the adequate precision metric. The ANOVA results for all investigated responses are summarized in Table 2.

The analysis demonstrated that most response models were statistically significant, including overrun (*p* = 0.0008), firmness (*p* = 0.0150), springiness (*p* = 0.0137), heat loss (*p* = 0.0011), and porosity (*p* = 0.0488). In contrast, the models for specific internal surface (*p* = 0.0896) and pore density (*p* = 0.0976) did not reach statistical significance at the 95% confidence level, although they remained close to the threshold, suggesting moderate model relevance.

To simplify the regression equations, the statistical significance of individual model terms was further evaluated (Table 3). Terms with *p* < 0.05 were considered statistically significant, whereas those with *p* > 0.10 were regarded as negligible and excluded from the final reduced models.

For overrun, sodium alginate (A) and Rügen chalk (B) exhibited highly significant effects (*p* = 0.0002 and *p* = 0.0017, respectively). In addition, interaction terms AB (*p* = 0.0352) and BC (*p* = 0.0200) were statistically significant, indicating that combined effects between formulation components contributed to gas incorporation. The Perlastan^®^–GDL factor (C) showed a marginal effect (*p* = 0.0989).

Firmness was primarily governed by sodium alginate, which was the only statistically significant factor (*p* = 0.002), while Rügen chalk (*p* = 0.6856) and Perlastan^®^–GDL (*p* = 0.9798) had no measurable influence within the studied range.

Springiness was significantly influenced by both sodium alginate (*p* = 0.0142) and Perlastan^®^–GDL (*p* = 0.0261), whereas Rügen chalk did not exhibit a statistically significant effect (*p* = 0.1981).

For heat loss, all three linear factors were significant (A: *p* = 0.0032; B: *p* = 0.0023; C: *p* = 0.0015). Additionally, interaction effects AC (*p* = 0.0241) and BC (*p* = 0.0197) were significant, indicating that both formulation composition and acidification-driven CO_2_ generation played a key role in thermal retention properties.

Regarding structural characteristics, sodium alginate significantly affected specific internal surface (*p* = 0.0104), porosity (*p* = 0.0087), and pore density (*p* = 0.0236). However, other factors showed limited or no statistical significance for these responses, with most *p*-values exceeding 0.05. Notably, quadratic terms for specific internal surface revealed that the curvature effect of factor C (*p* = 0.0428) was significant, while A^2^ approached significance (*p* = 0.0527), suggesting potential nonlinear behavior.

Across all responses, sodium alginate appeared most frequently among statistically significant terms, confirming its dominant role in determining both mechanical and structural foam properties.

To improve model parsimony and predictive performance, non-significant terms were removed, and reduced models were refitted (Table 4). The specific internal surface and pore density responses initially showed non-significant full models; however, after removal of non-significant terms, the reduced models became statistically significant (model *p* < 0.05), and these reduced models were used for subsequent interpretation. The refined models showed improved statistical significance for all responses, with model *p*–values ranging from 0.0003 (heat loss) to 0.0275 (pore density).

In the reduced models, sodium alginate remained a consistently significant factor across all responses. Rügen chalk retained significance for overrun (*p* = 0.0109) and heat loss (*p* = 0.0012), while Perlastan^®^–GDL contributed significantly to springiness (*p* = 0.0290) and heat loss (*p* = 0.0007). Interaction effects were largely eliminated, except for AC (*p* = 0.0169) and BC (*p* = 0.0135) in the heat loss model and BC (*p* = 0.0220) in the specific internal surface model. Additionally, quadratic terms A^2^ (*p* = 0.0070) and C^2^ (*p* = 0.0037) remained significant for specific internal surface, confirming nonlinear dependencies.

Model adequacy was further assessed using adjusted R^2^, predicted R^2^, and Adequate Precision values (Table 5). All models demonstrated Adequate Precision values above 4, indicating an adequate signal-to-noise ratio.

The heat loss model showed the best performance, with adjusted R^2^ = 0.8485 and predicted R^2^ = 0.7114 (ΔR^2^ = 0.14), indicating strong predictive capability. Overrun exhibited moderate predictability (adjusted R^2^ = 0.6299; predicted R^2^ = 0.4472; ΔR^2^ = 0.18), while firmness showed relatively good agreement between adjusted and predicted values (0.5618 vs. 0.4585; ΔR^2^ = 0.10). Springiness demonstrated slightly lower predictability (adjusted R^2^ = 0.4624; predicted R^2^ = 0.2699; ΔR^2^ = 0.19).

Structural responses showed more limited predictive performance. The specific internal surface exhibited good model quality (adjusted R^2^ = 0.7781; predicted R^2^ = 0.6292; ΔR^2^ = 0.14), whereas porosity (adjusted R^2^ = 0.4219; predicted R^2^ = 0.2899; ΔR^2^ = 0.13) and pore density (adjusted R^2^ = 0.2693; predicted R^2^ = 0.0720; ΔR^2^ = 0.19) demonstrated weaker predictive capability.

Importantly, ΔR^2^ values for all responses remained below 0.2, confirming acceptable agreement between adjusted and predicted coefficients of determination and supporting the validity of the developed models within the investigated design space.

### 2.12. Selection of the Optimal Formulation Based on Performance Criteria

The optimal formulation was selected using predefined performance thresholds. The selection criteria were established as follows: heat loss ≥ 120 s, firmness ≤ 10 N, and overrun ≥ 50%.

Experimental data were screened against these criteria, and only formulations meeting all three conditions were considered suitable. Among the tested compositions, formulation 7 fulfilled the defined requirements, exhibiting heat loss of 127 s, firmness of 7.4 N, and overrun of 57.1%.

**Figure 13:**
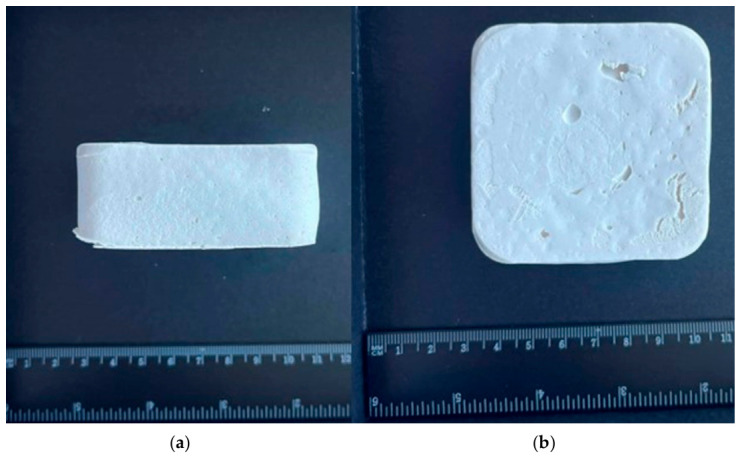
Representative foam specimen (Formulation 7) shown in side view (**a**) and top view (**b**).

Based on combined quantitative thresholds and qualitative assessment, formulation 7 was selected for further investigation (Figure 13).

Beyond compliance with quantitative thresholds, this formulation demonstrated favorable macroscopic handling properties. Qualitative evaluation during sample preparation and testing indicated cohesive structure, uniform surface morphology, and consistent elastic recovery after deformation compared with other tested formulations.

### 2.13. Thermal Conductivity

Thermal conductivity measurements were performed on the selected foam formulation (Formulation 7) and on a commercially available German natural healing mud (German: *Heilmoor*), which served as a reference material. Measurements were carried out under steady-state conditions using a symmetric heat flow meter apparatus in accordance with European standards for thermal insulation materials.

The measured thermal conductivity values were as follows: natural therapeutic peat (reference sample), 0.19325 W·m^−1^·K^−1^; optimized chalk–alginate foam (Formulation 7), 0.18657 W·m^−1^·K^−1^.

The selected foam exhibited slightly lower thermal conductivity than the reference mud, indicating improved thermal insulation capacity. Although the absolute difference between the materials is modest, the results demonstrate that the mineral–alginate foam is at least comparable to traditional therapeutic peloids in terms of heat retention potential, despite its substantially different microstructure and composition.

### 2.14. Cooling Time Analysis

**Figure 14:**
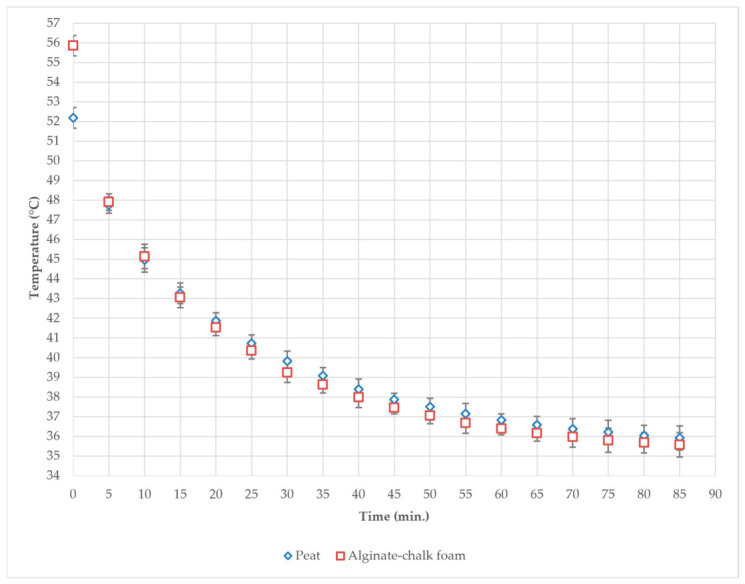
Cooling kinetics of natural therapeutic peat and Rügen chalk–alginate foam. Data represent mean core temperatures ± SD obtained from three independent experiments (*n* = 3) and recorded at fixed time intervals during passive cooling.

Cooling time analysis showed that the natural therapeutic peat cooled from 45 °C to 35 °C in approximately 80–85 min, while the optimized foam (Formulation 7) cooled over a very similar interval of about 75–80 min (Figure 14).

Although the foam initially reached a slightly higher peak temperature after preconditioning, both materials exhibited nearly identical cooling behaviour within the clinically relevant temperature window of 35–45 °C, and the cooling curves extended well beyond 60 min, so any impression of “60–min. cooling” would arise only from prematurely truncated analysis. The optimized foam displayed a marginally faster initial temperature drop, plausibly related to its porous structure and lower bulk density, but once the system entered the therapeutic range, the cooling rate stabilized and closely matched that of natural peat. Taken together with the thermal conductivity results, these data indicate that the optimized chalk–alginate foam (Formulation 7) provides thermal insulation comparable to traditional healing mud, maintains temperatures within the therapeutically effective range (35–45 °C) for more than one hour, and ensures controlled, gradual heat release compatible with safe skin contact, supporting its selection as a promising alternative to conventional peloids for external heat therapy applications.

### 2.15. Correlation Analysis of Formulation Parameters and Foam Properties

**Figure 15:**
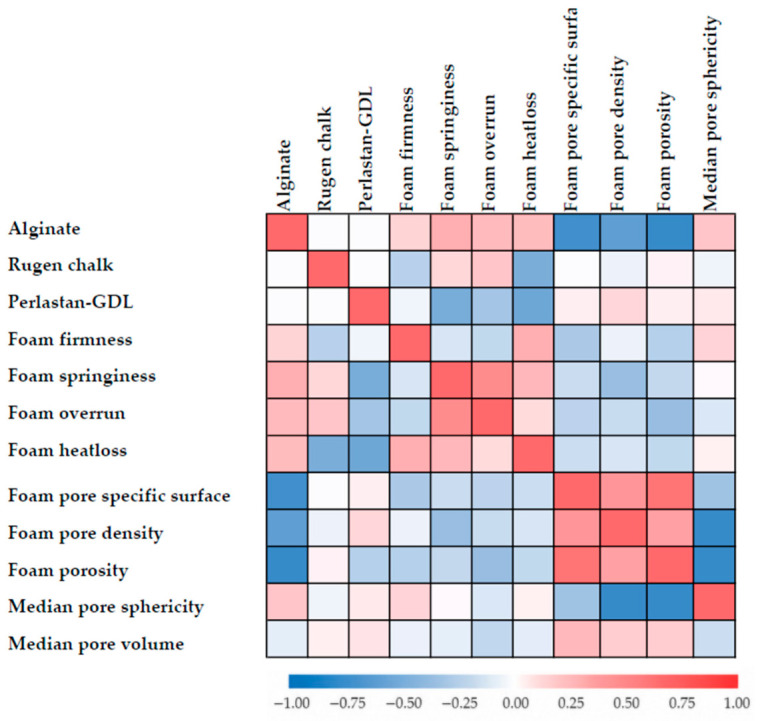
Heatmap illustrating Pearson correlation coefficients between formulation parameters and foam structural–mechanical properties. Red indicates positive correlations, and blue indicates negative correlations.

The correlation analysis revealed clear interdependencies between formulation components and the structural–mechanical properties of the Rügen chalk-based foams (Figure 15). Pearson correlation coefficients were calculated in SPSS (*n* = 15), and statistically significant relationships were identified at *p* < 0.05 and *p* < 0.01.

Sodium alginate exhibited several statistically significant correlations with both functional and structural properties. A positive correlation was observed with springiness (r = 0.551, *p* = 0.033), indicating that increasing alginate concentration enhances elastic recovery of the foam. In contrast, strong negative correlations were found with specific internal surface (r = −0.659, *p* = 0.008) and porosity (r = −0.684, *p* = 0.005), suggesting that higher alginate content leads to denser and less porous structures. Additionally, alginate showed a negative correlation with pore density (r = −0.567, *p* = 0.028), indicating a reduction in the number of pores per unit volume.

Rügen chalk did not exhibit statistically significant correlations with most responses (*p* > 0.05), although moderate trends were observed, such as a negative association with heat loss (r = −0.481, *p* = 0.069), suggesting a potential but limited influence on thermal behavior.

Perlastan^®^–GDL showed a significant negative correlation with heat loss (r = −0.519, *p* = 0.047), indicating that increased gas-generating and surfactant activity may accelerate heat dissipation. Other correlations involving this factor were not statistically significant.

Among the response variables, strong relationships were identified between mechanical and structural properties. Overrun showed a strong positive correlation with springiness (r = 0.772, *p* < 0.001), suggesting that foams with higher gas incorporation also exhibit greater elasticity. Firmness correlated positively with heat loss (r = 0.551, *p* = 0.033), indicating that more rigid structures may retain heat more effectively, although the present correlations do not establish a causal relationship.

µCT-derived structural parameters revealed particularly strong interrelationships. The specific internal surface was highly positively correlated with porosity (r = 0.944, *p* < 0.001) and pore density (r = 0.711, *p* = 0.003), confirming that increased pore number leads to greater internal surface development. Similarly, porosity was positively correlated with pore density (r = 0.634, *p* = 0.011) and median pore volume (r = 0.543, *p* = 0.036). In contrast, pore density showed a strong negative correlation with sphericity (r = −0.680, *p* = 0.005), indicating that highly porous structures tend to contain less regular (less spherical) pores.

Overall, the correlation analysis highlights sodium alginate as the dominant factor influencing both mechanical and structural foam properties, while the relationships among µCT parameters confirm that foam architecture is primarily governed by pore number and spatial distribution rather than pore size alone.

## 3. Discussion

This study demonstrates that an alginate-based solid foam incorporating Rügen chalk can be engineered to combine tunable mechanical properties, a well-defined porous microstructure, and heat retention characteristics comparable to traditional therapeutic muds used in balneology and spa medicine [2]. In line with previous reports on alginate hydrogels, foams, and composite sponges, the present findings confirm that the density and connectivity of the polymer network are key determinants of both mechanical behaviour and thermal performance [20,26]. By integrating a clinically used natural mineral into a solid-foam architecture, this work extends earlier approaches that mainly focused on synthetic or purely polymeric foams and provides a platform specifically tailored to thermotherapeutic applications.

An important aspect of the present formulation strategy is that the foam was not designed merely as a porous carrier but as a structured mineral–polymer system in which Rügen chalk participates both as a functional mineral phase and as a source of calcium ions for the alginate network’s formation. In the presence of GDL, gradual acidification promotes partial calcium carbonate dissolution, CO_2_ generation, and calcium-mediated alginate gelation. Thus, foaming and gelation occur simultaneously, allowing the mineral phase to be immobilized within the polymer matrix while generating a stable porous structure.

The correlation and response surface analyses consistently identified sodium alginate concentration as the dominant factor controlling foam firmness and springiness, whereas Rügen chalk acted as a secondary reinforcing filler and the Perlastan^®^–GDL system primarily modulated gas incorporation and gelation kinetics. This hierarchy is consistent with the view that alginate forms the load–bearing network and governs viscoelastic recovery, while mineral particles and surfactant-acid systems mainly fine-tune rigidity and structural stability [20,23,31]. The observation that excessive overrun reduces firmness aligns with published data on gas-expanded polymeric and gel foams, where excessive porosity and pore coalescence weaken the continuous phase. Although the predictive strength of the overrun model was moderate, it was sufficient to identify optimal formulation trends within the studied design space. In the present foams, intermediate overrun values and mineral loadings yielded a sponge–like microarchitecture with homogeneous pores, which corresponds to the most favorable combination of mechanical robustness and elasticity for topical application [32].

The dominant role of sodium alginate was further supported by both ANOVA and correlation analysis. Increasing alginate concentration increased firmness and springiness; however, it reduced the specific internal surface, porosity, and pore density. This indicates that higher alginate levels promote the formation of a stronger and more continuous gel matrix but simultaneously restrict bubble expansion and pore formation. Therefore, alginate acts as the main structural regulator of the system, balancing mechanical integrity against porosity development.

Microstructural analysis by optical microscopy and µCT confirmed the formation of a stable three-dimensional porous architecture generated by in situ CO_2_ evolution during alginate gelation. However, the µCT results showed that the foams were predominantly closed-cell rather than fully open-cell systems, with only localized pore coalescence and partial interconnectivity. This structure is advantageous for thermotherapeutic use, as closed-cell porosity can reduce convective heat loss while maintaining elasticity and low bulk density [33,34,35]. A gradual increase in pore size and total porosity with increasing overrun was observed, whereas formulations with intermediate expansion displayed the most structurally balanced microarchitecture. This supports a structure–function relationship in which polymer network density and controlled gas fraction jointly determine the macroscopic texture and mechanical behaviour of the foams [23,35].

The µCT-derived parameters also explain why differences in foam performance were not driven primarily by pore size alone. Median pore volume remained highly consistent across formulations, whereas specific internal surface, porosity and pore density varied more substantially. This suggests that the formulation’s composition mainly controlled the number and spatial distribution of pores rather than producing major shifts in individual pore size. Such behaviour is typical of gel-stabilized foams, where matrix viscosity and gelation kinetics limit bubble coarsening after initial gas generation.

Clinical and physicochemical studies on peloids emphasize that their therapeutic effect is strongly related to their ability to store heat and release it slowly, ensuring sustained thermal stimulation in combination with mineral and organic factors [2,36,37]. The present data show that the foam maintained temperatures within a typical therapeutic range (approximately 35–45 °C) for more than one hour, with only minimal differences relative to peat, suggesting that the new formulation can reproduce the essential physical component of peloid therapy while offering improved handling and reproducibility. The closed-cell character of the foam may also contribute to its favourable thermal behaviour. Entrapped gas within a hydrated mineral–polymer matrix can reduce effective thermal conductivity, while the water-rich alginate phase provides heat capacity and gradual heat release. This combination may explain why the selected foam achieved cooling behaviour comparable to natural therapeutic peat despite its lower density and different composition.

The pH of all formulations varied only within a narrow range around neutrality and was primarily governed by the interaction between alginate and Rügen chalk rather than by individual formulation variables. This behaviour is compatible with the known buffering capacity of calcium carbonate and the ion-binding properties of alginate. Given that Rügen chalk is already classified as a medical device for external use and has documented skin tolerability, the observed pH values support the suitability of the foam matrix for topical thermotherapeutic applications.

Compared with conventional mud or chalk packs, the solid foam format offers several practical advantages. It provides a defined shape, reduced messiness, easier handling, and potentially improved dose and application reproducibility. These characteristics may be particularly relevant for outpatient rehabilitation, home-based thermotherapy and institutional use, where hygienic handling, storage stability and standardized application are critical.

Several limitations should be acknowledged. First, this study focused on physicomechanical and thermal characterization under controlled laboratory conditions and did not include in vivo or clinical evaluations. Clinical studies on mud-pack and spa therapy indicate that clinical benefit depends not only on temperature and application time but also on complex neuroimmune and endocrine responses, as well as on the specific chemical and microbiological properties of peloids [37,38]. Whether the chalk–alginate foam can reproduce these biological effects, or whether it acts primarily as a physical heat carrier, remains to be clarified. Second, only one type of polymer–mineral system and a relatively narrow compositional space were investigated; exploring alternative polymers, crosslinkers, and mineral compositions could further optimize comfort, reusability, and drug-loading capacity [26,39]. Finally, long-term storage stability, microbiological robustness, and user acceptability under real-world conditions were not assessed and should be addressed in future work.

Future research should therefore include systematic in vitro and in vivo studies to evaluate skin tolerability, user comfort, and clinical efficacy of the chalk–alginate foam in indications where peloid therapy is already established, such as chronic low back pain, osteoarthritis, and myofascial pain syndromes [38,39]. In addition, the porous alginate–chalk matrix could be explored as a multifunctional platform combining thermotherapy with controlled topical delivery of anti-inflammatory or analgesic agents, building on the extensive literature on alginate-based drug delivery and dermal scaffolds [26,34,40]. Further work on scalability, packaging, and reheating protocols would facilitate translation from spa-like environments to home-based or outpatient settings. Together, these directions may help position alginate-based chalk foams as a promising complementary platform to traditional peloids, preserving their key thermotherapeutic advantages while overcoming limitations related to handling, hygiene, and batch-to-batch variability.

In addition, increasing environmental concerns related to the large-scale extraction and exploitation of natural peat deposits have stimulated interest in alternative thermotherapeutic materials with lower ecological impact [41]. This raises the question of whether mineral-based composites that rely on geologically stable carbonate resources could reproduce comparable thermophysical performance while reducing pressure on vulnerable peatland ecosystems.

To our knowledge, this is the first systematic integration of a clinically used natural chalk mineral into an alginate-based solid foam platform optimized via response surface methodology for thermotherapeutic application.

## 4. Materials and Methods

### 4.1. Peloid Material and Chemicals

Rügener Dreikronen Heilkreide^®^ (Kreidewerk Rügen GmbH, Bergen auf Rügen, Germany) was used as the mineral component of the foam formulations. The product is a commercially available medical-grade chalk intended for external therapeutic applications and is classified as a Class I medical device under Regulation (EU) 2017/745. It complies with relevant biocompatibility standards (EN ISO 10993-1, -5, -10) [42,43,44,45].

According to the manufacturer’s certificate of analysis, the material consists predominantly of calcium carbonate (CaCO_3_, 97.9%), with low moisture content (0.17%) and minimal coarse residue (>0.040 mm: 0.004%), indicating a highly refined and homogeneous mineral phase. The pH value of the material was 9.1, reflecting its mildly alkaline character.

The ionic composition is dominated by calcium (705 mg/L; 87.6% equivalent fraction) and hydrogen carbonate (2275 mg/L; 92.8% equivalent fraction), with minor contributions from magnesium, sodium, potassium, sulfate, chloride, and fluoride ions. Trace elements such as iron, manganese, and ammonium were present below detectable or negligible levels.

Sodium alginate (alginic acid sodium salt, CAS 9005-38-3; Tokyo Chemical Industry Co., Ltd. (TCI), Tokyo, Japan) was used as the primary biopolymeric matrix.

Perlastan^®^ SCG 50 HC MB (Schill & Seilacher “Struktol” GmbH, Hamburg, Germany), an amino acid-based anionic surfactant (INCI: Disodium Cocoyl Glutamate; CAS 68187-32-6, 55566-30-8), served as a foam stabilizer. It was used as supplied (≈50% active matter in aqueous solution) to facilitate air incorporation during mechanical mixing and to improve the stability of the CO_2_-generated porous structure during alginate gelation.

D–Glucono–δ–lactone (GDL; CAS 526-95-4, ≥99% purity; TCI Europe N.V., Zwijndrecht, Belgium) was used as a delayed acidifying agent to induce controlled gelation and in situ CO_2_ generation from chemical reaction with calcium carbonate.

Distilled water produced in the laboratory was used for all formulations and experiments. Natural therapeutic peat (Heilmoor; Schweriner Naturheil Pharmafit, Schwerin, Germany) was used as received for comparative thermal studies.

### 4.2. Experimental Design

A response surface methodology (RSM) based on a central composite design (CCD) was applied to evaluate the influence of formulation variables on the physico-mechanical and thermal properties of alginate-based Rügen chalk foams. Three independent factors were investigated: sodium alginate concentration (% *w*/*w*), Rügen chalk content (% *w*/*w*), and the Perlastan^®^ SCG 50 HC MB–GDL mixture (1:1, % *w*/*w*). A face-centred CCD (α = 1) comprising 15 experimental runs, including one centre point, was employed. The formulation compositions are presented in Table 6.

The following responses were evaluated: overrun (%), firmness (N), springiness (%), heat loss time (s), pH, specific internal surface (mm^−1^), porosity (%), mean pore volume (mm^3^), pore density (pores/mm^3^), and mean pore sphericity (–). Appropriate polynomial models were fitted to the data, and model adequacy was assessed using analysis of variance (ANOVA) at a significance level of *p* < 0.05. The adjusted R^2^, predicted R^2^, and adequate precision were used to evaluate model performance. Response surface and contour plots were generated using Design–Expert^®^ software (version 13, Stat–Ease Inc., Minneapolis, MN, USA) to visualize factor effects.

### 4.3. Preparation of Chalk–Alginate Foams (Mechanical Mixing Method)

Sodium alginate was gradually dispersed in distilled water at room temperature under continuous mechanical stirring (800 rpm) using an overhead stirrer to prevent particle agglomeration. Mixing was continued for 30 min until a visually homogeneous dispersion was obtained, which was then stored at 4 °C for 24 h to ensure complete polymer hydration and stabilization of rheological properties prior to further processing.

After full hydration, Rügen chalk and Perlastan^®^ SCG 50 HC MB were added sequentially to the alginate gel. Homogenization was performed in a 200 mL jar using a FagronLab™ PRO (UNGATOR^®^ PRO, PCCA, Houston, TX, USA) mixing system with a two-step program: an initial mixing step at 1.000 rpm for 160 s followed by a high-shear step at 1.200 rpm for 1.080 s.

Immediately after homogenization, GDL powder was added to the freshly prepared foam and gently mixed in the same jar at 500 rpm for 10 s to ensure uniform distribution while avoiding premature gelation. The resulting foams were kept at room temperature for 15 min and transferred into hermetically heat-sealed plastic bags to prevent dehydration, then left to gel under quiescent conditions at room temperature (22 ± 2 °C) for 24 h to achieve complete gelation and structural stabilization prior to physicochemical and mechanical testing.

### 4.4. Syringe-Based Foam Overrun Measurement

**Figure 16:**
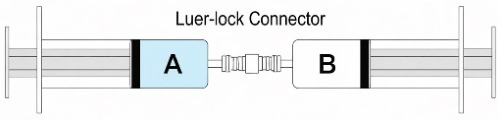
Overrun measurement. Schematic illustration of the experimental setup showing syringe A filled with the alginate–chalk–Perlastan^®^ mixture and syringe B containing GDL.

Foam expansion was additionally assessed using a dual–syringe mixing method described by Carugo et al. [46]. One syringe (A) was filled with the alginate–chalk–Perlastan^®^ mixture, while a second syringe (B), connected via a luer lock connector, contained GDL; the total mixture mass was 15 g (Figure 16). The formulation was transferred back and forth between the syringes for 10 cycles, inducing foam formation through turbulence and CO_2_ evolution. After mixing, the foam was left in syringe A and stored undisturbed for 24 h.

Foam overrun was calculated from syringe volume markings according to Formula (1):

(1) $$\mathrm{Overrun}\;\left(\%\right)=\frac{V_{\mathrm{after}}-V_{\mathrm{before}}}{V_{\mathrm{before}}}\times 100$$

where $V_{before}$ is the initial volume of the mixture, and $V_{\mathrm{after}}$ is the foam volume after mixing [33].

### 4.5. Texture Analysis (Firmness and Springiness)

Texture analysis was performed on alginate-based foam samples prepared using the FagronLab™ PRO (UNGATOR^®^ PRO) mixing system. Cylindrical specimens (5 cm diameter, 2 cm height) were cut from the bulk foams. Texture measurements were carried out using a TA.XTplusC Texture Analyzer (Stable Micro Systems, Godalming, UK) equipped with a flat 75 mm diameter compression platen.

Firmness was determined using a single-compression test in compression mode (distance), with a target distance of 8 mm, test speed of 1 mm·s^−1^, post-test speed of 10 mm·s^−1^, tare mode set to auto, and break mode off; firmness was defined as the maximum force recorded during this first compression cycle. Springiness was evaluated in a separate texture profile analysis test, in which the sample was subjected to two successive compressions under identical settings, and springiness was calculated as the ratio of height recovery between the first and second compression cycles. Due to minor, unavoidable variability in specimen dimensions, data from both tests were averaged across replicate measurements for each formulation.

### 4.6. Determination of pH

The pH values of the prepared foams were determined using a 766 Calimatic pH meter (Knick, Berlin, Germany) equipped with an SE 104 N puncture electrode. The instrument was calibrated before measurements using standard buffer solutions. The electrode was inserted directly into the foam matrix to obtain representative values for the semi-solid system.

### 4.7. Optical Microscopy of Foam Microstructure

Foam microstructure was examined by optical microscopy (40× total magnification) to qualitatively assess pore morphology and structural homogeneity. After complete gelation (24 h), samples were sectioned to obtain fresh cross-sections and imaged immediately to minimize deformation. Microscopy was performed using a Nikon Eclipse 50i optical microscope (Nikon Corporation, Tokyo, Japan) under identical settings with oblique illumination. Digital micrographs were acquired for qualitative morphological comparison and for correlation with foam expansion and mechanical properties; no quantitative image analysis was performed.

### 4.8. Micro-Computed Tomography (µCT) Evaluation of Foam Structure

For microstructural evaluation, cylindrical foam specimens were carefully cut from the bulk alginate–chalk foams. Investigations were performed using a RayScan 250E X-ray 3D computer tomograph (RayScan Technologies GmbH, Meersburg, Germany). A microfocus X-ray source with an output voltage ranging from 10 to 230 kV was used. The X-ray source irradiates the test object with a cone beam, and a flat panel detector with 2048 × 2048 pixels records 2D radiographic images. A total of 1890 projections were acquired as the object was rotated. Each projection was recorded with an integration time of 2 s and averaged 5 times. A voltage of 150 kV and a current of 150 µA were used, resulting in a focal spot of 26 µm. The spatial resolution achieved depended on the size of the investigated zone, resulting in voxel sizes ranging from 38 µm to 94 µm.

Quantitative parameters such as material volume, porosity, and surface area were extracted to characterize the structural heterogeneity and pore size distribution of the samples. The specific internal surface was calculated as the ratio of the total internal pore–matrix interface area to the material volume of the analysed specimen (ΣSurface/material volume), yielding a surface-to-volume ratio with units of mm^−1^. The porosity represents the fraction of segmented high-volume regions identified by voxel-based thresholding and is expressed as a percentage of the total sample volume.

The µCT data were segmented in VGStudio MAX 2023.3 (Volume Graphics GmbH, Heidelberg, Germany) using global grey-value thresholding (validated by visual slice inspection) followed by 3D region-growing and size/connectivity filters to clean noise and artefacts before extracting porosity and pore statistics.

### 4.9. Passive Heat Loss Test Under Ambient Cooling

Foam specimens from all 15 formulations were prepared as cylindrical discs (3 cm height and 2 cm thickness). A temperature probe was inserted into the geometric centre of each specimen and remained in place throughout the entire experiment. To prevent water uptake and structural damage during heating, each probe-equipped specimen was temporarily wrapped in a thin polyethylene film and immersed in a thermostatically controlled water bath (Fleischhacker Labortechnik, Schwerte, Germany) until the internal temperature reached 45 °C. Immediately after removal from the water bath, the polyethylene film was carefully removed while keeping the probe in position, and the unwrapped specimens were placed under ambient laboratory conditions, where the natural cooling process was monitored. Core temperature was measured using a PC 8+ DHS Bench Meter (XS Instruments, Modena, Italy), and the time required for the internal temperature to decrease from 45 °C to 35 °C was recorded and defined as the heat loss time in seconds.

### 4.10. Thermal Conductivity Measurement

**Figure 17:**
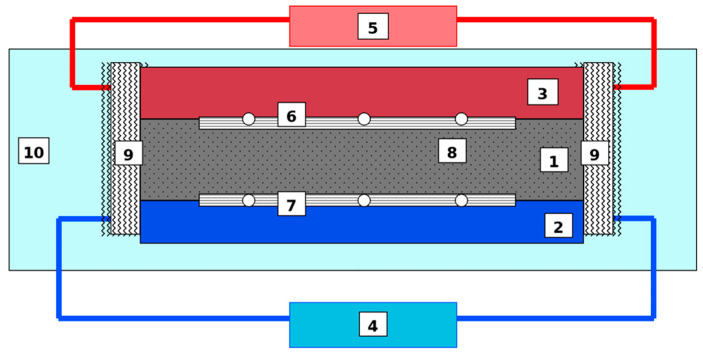
Schematic diagram of the heat flow meter measurement system. 1—A test specimen; 2—cooling plate; 3—heating plate; 4—cooling thermostat; 5—heating thermostat; 6—heat flow meter to the heating panel; 7—heat flow meter to the cooling plate; 8—thermocouple; 9—protected space; 10—environment with controlled constant temperature.

The thermal conductivity of the selected dermatological foam formulation was measured using a FOX 600 heat flow meter apparatus (LaserComp, Saugus, MA, USA). Measurements were performed in accordance with the EN 12667 standard for steady-state thermal conductivity determination [47]. A schematic diagram of the measurement setup is shown in Figure 17.

During the measurements, foam specimens with dimensions of 200 × 200 × 30 mm were tested, whereas the central measuring area of the apparatus was 250 × 250 mm. As the minimum required specimen size for the method is 600 × 600 × 30 mm, an extruded polystyrene (XPS) board was used as a frame for the test specimen. First, the thermal conductivity of the XPS board was determined using a full-size XPS specimen (600 × 600 × 30 mm). Subsequently, a 200 × 200 mm cavity was cut in the center of the XPS board and filled with the therapeutic foam (or peat) sample.

After performing thermal conductivity measurements on the composite (foam sample + XPS frame), the thermal conductivity coefficient of the foam sample, $\lambda_{\mathrm{spec}}$ (W·m^−1^·K^−1^), was calculated according to Formula (2):

(2) $$\lambda_{\mathrm{spec}}=\frac{\lambda_{\mathrm{sum}}A_{\mathrm{sum}}-\lambda_{\mathrm{xps}}A_{\mathrm{xps}}}{A_{\mathrm{spec}}}$$

where $\lambda_{\mathrm{spec}}$ is the thermal conductivity coefficient of the foam sample (W·m^−1^·K^−1^), $\lambda_{\mathrm{sum}}$ is the overall thermal conductivity coefficient of the foam sample plus XPS frame (W·m^−1^·K^−1^), $\lambda_{\mathrm{xps}}$ is the thermal conductivity of the XPS frame (W·m^−1^·K^−1^), $A_{\mathrm{sum}}$ is the total area of the XPS frame with the foam sample (m^2^), $A_{\mathrm{xps}}$ is the area of the XPS frame without the foam sample (m^2^), and $A_{\mathrm{spec}}$ is the area of the foam specimen (m^2^).

### 4.11. Thermal Release Test Under Controlled Temperature Gradient

Following the thermal conductivity measurements, a thermal release (heat dissipation) test was performed on the selected foam formulation and reference peat using the same symmetric heat flow meter apparatus. During heating and preconditioning, foam specimens were wrapped in polyethylene film to prevent moisture loss and changes in water content. Foam specimens (200 × 200 × 30 mm) were preconditioned in a laboratory oven at 75 °C for 4.5 h to ensure a uniform internal temperature.

The samples were then placed between the plates of the apparatus, with the upper plate maintained at 20 °C to simulate ambient room temperature and the lower plate maintained at 36 °C to mimic the surface temperature of human skin. A thermocouple was positioned at the lower interface between the specimen and the 36 °C plate. Temperature readings were recorded at 5 min intervals until the foam core temperature decreased from 50 °C to 35 °C, and the corresponding time was defined as the thermal release time. For comparison, a natural therapeutic peat specimen of identical dimensions was tested under the same conditions.

### 4.12. Statistical Analysis

Experimental data were analyzed using Design–Expert^®^ software (Stat–Ease Inc., Arden Hills, MN, USA). Response surface models were fitted, and analysis of variance (ANOVA) was applied to evaluate the significance of main effects and factor interactions. Nonsignificant terms were removed stepwise while respecting model hierarchy, and the ANOVA statistics models. Optimization was performed using a desirability function approach to identify formulations that balanced mechanical performance, foam expansion, and thermal behaviour. In addition, statistical data processing and descriptive analyses were carried out in SPSS Statistics (IBM Corp., Armonk, NY, USA) to ensure standardized data handling and result validation. Pearson correlation coefficients were calculated in SPSS 31 (IBM Corp., Armonk, NY, USA) to assess relationships between formulation variables and response parameters. Correlation strength was interpreted as weak (|r| < 0.3), moderate (0.3 ≤ |r| < 0.7), or strong (|r| ≥ 0.7). A heatmap was constructed to visualize correlation interdependencies. All experiments were performed in triplicate, and data are expressed as mean (standard deviation).

### 4.13. Ethical Statement and Generative AI Disclosure

This study did not involve human participants or animals and therefore did not require ethical approval. Generative artificial intelligence tools were used solely to assist with language refinement and manuscript structuring. All experimental design, data collection, statistical analyses, and data interpretation were performed by the authors.

## 5. Conclusions

In conclusion, this study demonstrates that Rügen chalk can be successfully integrated into an alginate-based solid foam to create a semi-solid porous system that combines a high natural mineral load with a defined, convenient, and reproducible dosage form. By coupling the intrinsic calcium carbonate content of Rügen chalk with glucono–δ–lactone-mediated acidification, the mineral phase simultaneously serves as a therapeutic component and as a source of calcium ions and CO_2_ for in situ gelation, foam formation, and stabilization. Sodium alginate was identified as the dominant factor governing foam structure, firmness, elasticity, porosity, and pore density, while the Perlastan^®^–GDL system enabled controlled foaming and gelation kinetics. Microcomputed tomography revealed a predominantly closed-cell porous architecture composed mainly of fine pores, with structural differences between formulations determined primarily by pore number and spatial distribution rather than pore size. The selected formulation exhibited thermal conductivity and heat-retention properties comparable to therapeutic peat, maintaining clinically relevant temperatures for more than one hour. Overall, the developed chalk–alginate foam combines effective mineral loading, elastic porosity, and sustained heat retention, making it a promising platform for balneotherapy, rehabilitation, and home-based thermotherapy applications, while providing a foundation for further formulation optimization and future translational studies.

## Figures and Tables

**Table 1:** Quantitative µCT-derived structural parameters of all formulations.

Formulation Code	Specific Internal Surface (mm^−1^)	Porosity (%)	Mean Pore Volume (mm^3^)	Pore Density (Pores/mm^3^)	Mean Pore Sphericity (–)
F1	0.61	4.43	0.06	0.92	0.80
F2	0.91	7.52	0.06	1.40	0.79
F3	0.47	3.85	0.05	0.67	0.90
F4	0.60	4.66	0.05	0.96	0.87
F5	0.50	4.45	0.05	0.75	0.88
F6	0.61	5.84	0.06	0.73	0.88
F7	0.86	7.53	0.06	0.85	0.84
F8	0.47	4.16	0.05	0.63	0.90
F9	0.51	4.83	0.05	0.78	0.83
F10	1.03	7.58	0.06	1.05	0.88
F11	0.37	2.82	0.04	0.77	0.84
F12	0.63	4.85	0.05	1.07	0.84
F13	0.55	5.46	0.05	0.97	0.84
F14	0.45	3.83	0.05	0.76	0.87
F15	0.42	3.93	0.06	0.72	0.90

**Table 2:** ANOVA data of the fit of the model.

Response	Selected Model	Model *p*-Value
Overrun (%)	(2FI)	0.0008
Firmness (N)	Linear	0.0150
Springiness (%)	Linear	0.0137
Heat loss (s)	(2FI)	0.0011
Specific internal surface (mm^−1^)	Quadratic	0.0896
Porosity (%)	Linear	0.0488
Pore density (pores/mm^3^)	(2FI)	0.0976

**Table 3:** Statistical significance (*p*-values) of main factors and interaction terms for the evaluated foam properties.

Response							
Factors	Overrun	Firmness	Springiness	Heat Loss	Specific Internal Surface	Porosity	Pore Density
A (sodium alginate)	*p* = 0.0002	*p* = 0.002	*p* = 0.0142	*p* = 0.0032	*p* = 0.0104	*p* = 0.0087	*p* = 0.0236
B (Rügen chalk)	*p* = 0.0017	*p* = 0.6856	*p* = 0.1981	*p* = 0.0023	*p* = 0.9797	*p* = 0.6005	*p* = 0.7629
C (Perlastan^®^–GDL)	*p* = 0.0989	*p* = 0.9798	*p* = 0.0261	*p* = 0.0015	*p* = 0.5488	*p* = 0.5181	*p* = 0.2262
AB	*p* = 0.0352	–	–	*p* = 0.7551	*p* = 0.7553	–	*p* = 0.2365
AC	*p* = 0.1822	–	–	*p* = 0.0241	*p* = 0.6325	–	*p* = 0.0962
BC	*p* = 0.02	–	–	*p* = 0.0197	*p* = 0.0938	–	*p* = 0.2694
A^2^	–	–	–	–	*p* = 0.0527	–	–
B^2^	–	–	–	–	*p* = 0.8578	–	–
C^2^	–	–	–	–	*p* = 0.0428	–	–

**Table 4:** ANOVA data of the reduced models after removal of non-significant factors.

Response							
	Overrun	Firmness	Springiness	Heat Loss	Specific Internal Surface	Porosity	Pore Density
**Model *p*-value**	0.0010	0.0008	0.0096	0.0003	0.0005	0.0052	0.0275
**Factors**							
A (sodium alginate)	*p* = 0.0015	*p* = 0.0008	*p* = 0.0158	*p* = 0.0018	*p* = 0.0004	*p* = 0.0052	*p* = 0.0275
B (Rügen chalk)	*p* = 0.0109	–	–	*p* = 0.0012	–	–	–
C (Perlastan^®^–GDL)	–	–	*p* = 0.0290	*p* = 0.0007	–	–	–
AB	–	–	–	–	–	–	–
AC	–	–	–	*p* = 0.0169	–	–	–
BC	–	–	–	*p* = 0.0135	*p* = 0.0220	–	–
A^2^	–	–	–	–	*p* = 0.0070	–	–
B^2^	–	–	–	–	–	–	–
C^2^	–	–	–	–	*p* = 0.0037	–	–

**Table 5:** Statistical parameters used to assess the adequacy of the reduced models.

Response	Adjusted R^2^	Predicted R^2^	Δ R^2^	Adeq Precision
Overrun (%)	0.6299	0.4472	0.18	10.0
Firmness (N)	0.5618	0.4585	0.10	7.5
Springiness (%)	0.4624	0.2699	0.19	7.5
Heat loss (s)	0.8485	0.7114	0.14	15.7
Specific internal surface (mm^−1^)	0.7781	0.6292	0.14	11.2
Porosity (%)	0.4219	0.2899	0.13	5.8
Pore density (pores/mm^3^)	0.2693	0.0720	0.19	4.2

**Table 6:** Formulation matrix of alginate-based Rügen chalk foams used in the central composite design (CCD).

Formulation Code	Water (wt.%)	Sodium Alginate (wt.%)	Rügen Chalk (wt.%)	Perlastan^®^ (wt.%)	D–Glucono–δ–Lactone (wt.%)
F1	57.0	3.5	32.5	35	3.5
F2	54.5	2.5	35.0	4.,0	4.0
F3	55.5	3.5	35.0	3.0	3.0
F4	59.5	2.5	30.0	4.0	4.0
F5	56.5	3.0	32.5	4.0	4.0
F6	55.0	3.0	35.0	3.5	3.5
F7	61.5	2.5	30.0	3.0	3.0
F8	53.5	3.5	35.0	4.0	4.0
F9	56.5	2.5	35.0	3.0	3.0
F10	58.0	2.5	32.5	3.5	3.5
F11	58.5	3.0	32.5	3.0	3.0
F12	60.0	3.0	30.0	3.5	3.5
F13	57.5	3.0	32.5	3.5	3.5
F14	60.5	3.5	30.0	3.0	3.0
F15	58.5	3.5	30.0	4.0	4.0

## Data Availability

The original contributions presented in this study are included in the article. Further inquiries can be directed to the corresponding author.

## Abbreviations

The following abbreviations are used in this manuscript:

GDL	D–glucono–δ–lactone;
µCT	Microcomputed tomography;
RSM	Response surface methodology;
CCD	Central composite design;
ANOVA	Analysis of variance;
CaCO_3_	Calcium carbonate;
XPS	Extruded polystyrene;
INCI	International Nomenclature of Cosmetic Ingredients;
CO_2_	Carbon dioxide.
